# COTE1 Facilitates Intrahepatic Cholangiocarcinoma Progression via Beclin1-Dependent Autophagy Inhibition

**DOI:** 10.1155/2023/5491682

**Published:** 2023-09-22

**Authors:** Hai Zhang, Shu Chen, Sanrong Xu, Xiangcheng Li

**Affiliations:** ^1^Key Laboratory on Living Donor Transplantation, Ministry of Public Health, Department of Liver Transplantation Center, The First Affiliated Hospital of Nanjing Medical University, Nanjing 210029, China; ^2^Department of Hepatobiliary Surgery, The Affiliated Hospital of Jiangsu University, Zhenjiang 212001, China

## Abstract

COTE1 was recently described as an oncogene in hepatocellular carcinoma and gastric cancer. However, the roles of COTE1 in intrahepatic cholangiocarcinoma (ICC) are little known. Our study is aimed at clarifying novel functions of COTE1 in ICC progression, including proliferation, invasion, and autophagy. By using quantitative real-time PCR, immunohistochemistry staining, and western blotting, we found that COTE1 expression was frequently upregulated in ICC tissues, compared to paracarcinoma tissues. High COTE1 expression was significantly correlated with aggressive clinical features and predicted poor prognosis of ICC patients. Functional experiments revealed that ectopic COTE1 expression promoted ICC cell proliferation, colony formation, cellular invasion, migration, and in vivo tumorigenicity; in contrast, COTE1 knockdown resulted in the opposite effects. At molecular mechanism in vitro and vivo, our study revealed that COTE1 overexpression suppressed autophagy via Beclin1 transcription inhibition; conversely, COTE1 silencing facilitated autophagy through promoting Beclin1 expression. Furthermore, the suppression of COTE1 knockdown on cellular growth and invasion was rescued/aggravated by Beclin1 inhibition/accumulation. Our data, for the first time, illustrate that COTE1 is an oncogene in ICC pathogenesis, and the ectopic COTE1 expression promotes ICC proliferation and invasion via Beclin1-dependent autophagy inhibition.

## 1. Introduction

Intrahepatic cholangiocarcinoma (ICC), a highly aggressive malignant tumor originating from intrahepatic bile duct represents the second most common hepatic malignancy accounting for approximately 10-20% of all diagnosed liver cancers [1]. Its incidence and mortality are rising globally over the past decades, especially in the east countries, including China [2]. Although radical hepatic resection is considered as the potentially curative approach for patients at early stage, the prognosis remains poor due to the high incidence of recurrence and metastasis [3]. Therefore, a better understanding of the molecular mechanisms underlying ICC progression would be necessary to obtain effective therapeutic strategies for patients.

Autophagy is an intracellular tightly orchestrated process involved in degradation of damaged organelles and misfolded or mutated proteins [4]. This self-digestion system was found to maintain cellular homeostasis of normal cells and regulate progression of a series of diseases, including microbial invasion, neurodegeneration, and cancer [5–7]. The role of autophagy in cancer biology is dichotomous. LC3, an important regulator of autophagy, was found aberrant overexpressed in several solid tumors and predicted poor prognosis [8, 9]. However, Beclin1, another dominant monitor of autophagy, was discovered abnormally downregulated in multiple cancers and acted as a tumor suppressor [10, 11]. The Beclin1-mediated autophagy was frequently modulated by certain oncogenes, such as TRIM59, COPS3, and HER2, either through regulating the transcription, ubiquitination, or phosphorylation of Beclin1 [12–15].

COTE1 is located in chromosome 1q21 [16], which was considered to be one of the most frequently amplified regions in tumor [17]. The amplification of 1q21 target oncogenes was supposed to be closely associated with aggressive progression and inferior outcomes in malignancies [18, 19]. As a little-studied gene, the biological function of COTE1 remains ambiguous. Previous research found that expression of COTE1 correlated with activation of endogenous SREBP-1 (sterol-regulatory element binding protein) *in vitro* and speculated that it plays a role in lipid metabolism [20]. Our previous research demonstrated that COTE1 functions as an oncogene in HCC. Ectopic overexpression of COTE1 promoted HCC cell invasion [21]; besides, upregulation of COTE1 could physically interact with WW domain-containing oxidoreductase (WWOX), induce WWOX dephosphorylation, subsequently resulting in WWOX-mediated mitochondrial apoptosis suppression and cell cycle progression stimulation [22]. Recently, Wu et al. [23] identified the upregulation of COTE1 protein and mRNA in gastric cancer (GC) and discovered the prognostic value of high COTE1 expression in patients with GC and further predicted the potential involved signaling pathway from the Kyoto Encyclopedia of Genes and Genomes (KEGG) and Gene Ontology (GO) enrichment analyses.

In the current study, we systematically investigated the expression of COTE1 in ICC specimens and cell lines and analyzed the correlation between COTE1 expression and clinical characteristics, including overall survival and recurrence. We then explored the functional roles of COTE1 in ICC cell growth, invasion, and autophagy in vitro and in vivo. Our collective data indicated that COTE1 could contribute to progression of ICC through Beclin1-dependent autophagy regulation.

## 2. Materials and Methods

### 2.1. Patients and Follow-Up

A total of 58 patients diagnosed with ICC at the Affiliated Hospital of Jiangsu University (Zhenjiang, China) from January 2005 to December 2010 were enrolled in this study. All patients had undergone curative resection, and all tumor specimens were histologically confirmed by a pathologist. The patients had regular follow-up after surgical treatment until May 2020. This study was approved by the Ethics Committee of Affiliated Hospital of Jiangsu University.

### 2.2. Cell Lines

The human cholangiocarcinoma cell lines RBE, HuCCT1, and human intrahepatic biliary epithelial derived noncancer cells (HIBEpiC) were purchased from Chinese Academy of Sciences Cell Bank (Shanghai, China). All cell lines were cultured in RPMI-1640 (GIBCO, Hangzhou, China) containing 10% fetal bovine serum in a humidified incubator at 37°C with 5% CO_2_.

### 2.3. Quantitative Real-Time Polymerase Chain Reaction (qRT-PCR) and Semiquantitative Reverse Transcription-Polymerase Chain Reaction (RT-PCR)

Total RNA from clinical tissues and cultured cells was extracted with TRIzol (Invitrogen, Carlsbad, CA) and was used to synthesize cDNA with a M-MLV reverse transcriptase kit (Promega, Madison, USA). For qRT-PCR, the TaKaRa PCR Thermal Cycler Dice Detection System and SYBR green dye (TaKaRa, Otsu, Japan) were used according to the instructions recommended by the manufacturer. For RT-PCR, a total of 2 mg RNA was added into a 20 mL reaction, and the products were observed on a 2% agarose gel by electrophoresis. The expression of mRNAs was normalized to that of *β*-actin. The primers used in the experiment are listed in Supplementary Table S1.

### 2.4. Immunohistochemistry Staining and Evaluation

Tumor tissues were formalin-fixed, paraffin-embedded, and cut into 4 mm sections. The sections were deparaffinized in xylene, rehydrated with graded ethanol, and repaired by ethylenediaminetetraacetic acid (pH 8.0). The thickness was soaked in 3% H_2_O_2_ to quench the peroxidase activity in tissues. Slides were incubated in normal goat serum to block nonspecific antibody. Then, the sections were incubated with primary antibodies at 4°C for 12 hours. After washing with PBS buffer for three times, the sections were subsequently incubated with secondary antibodies at 37°C for 2 hours. Finally, the thicknesses were stained with DAB reagent (Maixin Bio. Ltd., Fuzhou, China). The samples were observed under a light microscope. The primary antibodies used in this study were as follows: goat anti-COTE1 antibody (1 : 100; Santa Cruz Biotechnology, CA, USA) and rabbit anti-Beclin-1 antibody (1 : 100; Santa Cruz Biotechnology, CA, USA).

Immunostaining analysis was performed in a blind manner by two independent pathologists. The expression level of COTE1 and Beclin-1 was evaluated by integrating the percentage of positive staining cells in the whole core (negative: score 0, weak: score 1, moderate: score 2, and strong: score 3) and the intensity of positive staining (<5%: score 0, 5-25%: score 1, 25-50%: score 2, 50-75%: score 3, and >75%: score 4). The final expression score was recorded by using an immunoreactive score (IRS), the product of positivity, and intensity score. The IRS value≦6 score was defined as “low expression” that >6 score was considered as “high expression” [24].

### 2.5. Western Blotting Analysis

Western blotting analysis was performed as described previously [22]. Antibodies used in this experiment were as follows: goat anti-COTE1 (1 : 200; Santa Cruz Biotechnology, CA, USA), rabbit anti-LC3A/B antibody (1 : 1000; Cell Signaling Technology, Massachusetts, USA), mouse anti-P62 antibody (1 : 1000; Cell Signaling Technology, Massachusetts, USA), rabbit anti-Beclin-1 antibody (1 : 200; Santa Cruz Biotechnology, CA, USA), and anti-*β*-actin (1 : 500; Santa Cruz Biotechnology, CA, USA). The gray value of proteins was quantitatively analyzed by ImageJ (v.1.8.0).

### 2.6. Transfection

Cells were transfected with small interfering RNA (siRNA) against COTE1 (siCOTE1) and Beclin-1 (siBeclin-1), short hairpin RNA (shRNA) against COTE1 (shCOTE1) and Beclin-1 (shBeclin-1), and plasmid expressing COTE1 (pcDNA3.1B-FLAG-GFP-COTE1) and Beclin-1 (pcDNA3.1-Beclin-1) using Lipofectamine 2000 (Invitrogen, CA, USA) according to the manufacturer's instruction. The siRNA, shRNA against COTE1, and plasmid expressing COTE1 were designed and synthesized as reported in our previous article [22]. The siRNA, shRNA against Beclin-1, and plasmid expressing Beclin-1 were designed and constructed by GenePharma Co. (Shanghai, China). The sequences of siRNAs/shRNAs used in this study are listed in Supplementary Table S1.

### 2.7. Construction of Stable Cells

Clones stably overexpressed COTE1 or silencing COTE1 were constructed according our previous research [22]. Briefly, COTE1 plasmid and oligonucleotides for shRNA were, respectively, cloned into pcDNA3.1B-FLAG-GFP (Chinese National Human Genome Center, Shanghai, China) and pGCsi-H1_Neo_GFP (Chinese National Human Genome Center, Shanghai, China) vectors, both of which contain GFP report gene and neomycin resistance gene. Stably cells were selected for 2 weeks using G418 (800 mg/mL), and the transfection efficacy was determined by immunofluorescence.

### 2.8. Cell Proliferation and Colony Formation

For cell growth curve, a Cell Counting Kit-8 (CCK-8, Dojindo Laboratories, Kumamoto, Japan) was used to measure cell viability. For plate colony formation, transfected cells were cultured in fetal bovine serum containing G418 (800 mg/mL) for 2-3 weeks. Clones were stained with Coomassie Brilliant Blue R-250 (CBBR-250). For soft agar colony formation, cells were plated into 24-well plates containing 1% base agar and 0.5% top agar and incubated for 3 weeks until the colonies could be counted under a dissecting microscope.

### 2.9. Cell Invasion Assay

For cell invasion assay, the 24-well transwells (8 mm pore size; BD Biosciences, San Jose, CA), coated with Matrigel, were used. A total of 1 × 10^5^ cells were suspended in serum-free medium in the top chamber, while medium containing 10 mg fibronectin and 10% FBS was added into the bottom chamber. After incubated for 48 hours, the translocated cells were fixed in 4% paraformaldehyde and stained with 0.5% crystal violet and observed under a microscope. For quantification, the average number of invasive cells in five fields (left, right, upper, lower, and middle) was applied.

### 2.10. Wound-Healing Assay

For cell migration, the wound-healing assay was performed. The transfected cells were seeded in 6-well plate and grown to 80-90% confluence. A wound was created by dragging a sterile pipette tip across the cell surface. The wounded areas were observed and recorded for incubations of 0, 24, 48, and 72 hours. For quantification, the wounded areas were calculated by ImageJ (v.1.8.0), and the migration rate at different time points was measured by using the following formula: [(wounded area 0 h − wounded area n h)/wounded area 0 h] × 100% (*n* = 24, 48, and 72).

### 2.11. Immunofluorescence Staining

The immunofluorescence assay was performed to detect the accumulation of LC3 II, a marker of autophagosome formation. Briefly, cells were prepared on glass coverslips and fixed with 4% paraformaldehyde for 20 min. The fixed cells were blocked with phosphate-buffered saline (PBS) containing 10% bovine serum albumin (BSA) for 30 min and incubated with primary antibody (anti-LC3A/B, 1 : 100) overnight. Then, cells were incubated in specific secondary antibody (1 : 100) for 1 h and finally stained with 4′,6-diamidno-2-phenylindole (DAPI) before observation. Immunofluorescence was evaluated using a confocal microscope (Olympus, USA) and quantized by ImageJ (v.1.8.0).

### 2.12. Transmission Electron Microscopy (TME)

TME was carried out to observe the autophagosomes in ICC cells. In brief, cells were fixed with 2% glutaraldehyde in 0.1 mol/L PBS and postfixed with 2% OsO_4_ buffer. Next, cells were dehydrated in a grade of ethanol and embedded in Araldite. Ninety nanometer ultrathin sections were double-stained with 1% uranyl acetate and 0.2% lead citrate. The images were captured using an electron microscope (JEM. 1010; JEOL, Tokyo, Japan).

### 2.13. In Vivo Tumor Growth Assays

To construct xenograft models, 4-week-old male BALB/c nude mice were used in this study. 3 × 10^6^ stable cells with COTE1 alteration were subcutaneously injected near the scapulas of nude mice. The sizes were monitored per 7 days once the tumors formed. The volume was measured using the following formula: 0.5 × length × width^2^ (mm^3^). The mice were sacrificed at day 28, and the tumors were weighed.

### 2.14. Data Analysis and Statistics

All experiments were repeated independently three times. Statistical analyses were performed using SPSS 24.0 software (SPSS Inc., IL, USA). Student's *t*-test was used for comparison of quantitative variables, Chi-square test or Fisher's exact test was performed to evaluate the differences of categorical data. Pearson's correlation analysis was used to determine correlations in protein expression between COTE1 and Beclin-1. The prognostic significance was determined by the Kaplan-Meier analysis and log-rank tests. Univariate and multivariate were conducted by the Cox's proportional hazard regression model. All experiments were performed at least three times, and statistical significant differences were defined as ^∗^*P* < 0.05, ^∗∗^*P* < 0.01, and ^∗∗∗^*P* < 0.001.

## 3. Results

### 3.1. COTE1 Was Upregulated in ICC

**Figure 1:**
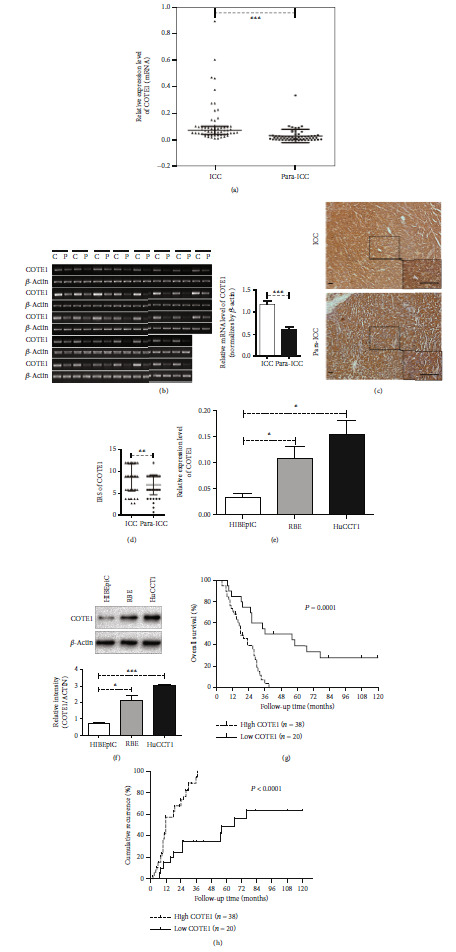
High COTE1 expression in intrahepatic cholangiocarcinoma (ICC) and predicts a poor prognosis. (a) COTE1 mRNA expression in ICC tumor tissues (*n* = 58) and paracarcinoma tissues (*n* = 58) were determined by qRT-PCR; data are presented as median with interquartile rage (^∗∗∗^*P* < 0.001). (b) Representative results of COTE1 overexpression in ICC specimens by RT-PCR (*n* = 38). The bands of nucleic acid electrophoresis were quantized by ImageJ (mean ± SD), and the relative level of COTE1 was normalized to that of *β*-actin (^∗∗∗^*P* < 0.001). (c, d) COTE1 protein expression in ICC (*n* = 58) and paracarcinoma tissues (*n* = 58) were measured by IHC (original magnification, ×100, low right image, ×400; bar = 10 *μ*m), and the immunoreactive score (IRS) was statistically analyzed by SPSS (mean ± SD; ^∗∗^*P* < 0.01). (e, f) mRNA and protein of COTE1 in ICC cell lines (RBE, HuCCT1) and human intrahepatic biliary epithelial-derived noncancer cells (HIBEpiC) were detected by qRT-PCR and WB (mean ± SD; ^∗^*P* < 0.05, ^∗∗∗^*P* < 0.001). (g, h) The cumulative incidence of overall survival and recurrence in ICC patients with high (*n* = 38, *P* = 0.0001) vs. low (*n* = 20, *P* < 0.0001) COTE1 expression.

To investigate the expression of COTE1 in ICC, we first detected the mRNA level of COTE1 in 58 pairs of ICC and peritumoral specimens through qRT-PCR. The results revealed that COTE1 was significantly upregulated in tumor samples compared with adjacent tissues (38/58; *P* < 0.001; Figure 1(a)). Then, we validated the observation in the 38 pairs of specimens with high COTE1 expression by using RT-PCR (*P* < 0.001; Figure 1(b)). Moreover, we assessed the protein level of COTE1 through IHC in the matched ICC and adjacent nontumor tissues and confirmed the similar findings (*P* < 0.01; Figures 1(c) and 1(d)). Additionally, we performed qRT-PCR and western blotting analyses to examine the mRNA and protein expressions of COTE1 in available ICC cell lines, RBE and HuCCT1. Compared with HIBEpiC, normal healthy human intrahepatic biliary epithelial cells, the expression of COTE1 was considerably elevated in ICC cells (*P* < 0.05; Figure 1(e); *P* < 0.001; Figure 1(f)). The resulting data indicated that the expression of COTE1 was significantly increased in ICC tumor tissues and cell lines.

### 3.2. High COTE1 Expression Predicts Poor Prognosis in ICC

To elucidate the correlation between COTE1 expression and clinicopathological characteristics of ICC patients, we carried out IHC to obtain the IRS of COTE1 in tumor tissues. As shown in Table 1, patients with high COTE1 IRS were more likely to have elevated carcinoembryonic antigen (CEA) level (*P* = 0.037), worse histologic differentiation (*P* = 0.003), lymphatic metastasis (*P* < 0.001), vascular invasion (*P* = 0.012), and multiple focus (*P* = 0.027). However, other clinical features appeared to have only a slight association with the expression of COTE1 (*P* > 0.05). Next, we assessed the prognostic value of COTE1 expression in ICC. The survival analysis showed that the 1-, 3-, and 5-year survival rates in the low COTE1 expression group were 85.00%, 50.00%, and 38.89%, respectively. In contrast, patients with high COTE1 expression displayed 1-, 3-, and 5-year survival rates of 71.05%, 3.72%, and 0%, respectively, which were much worse than the counterparts (*P* = 0.001; Figure 1(g)). The cumulative recurrence incidence of patients with high COTE1 expression at 1, 3, and 5 years (57.90%, 94.83%, and 100.00%, respectively) was obviously higher (*P* < 0.001; Figure 1(h)) than those of patients with low COTE1 expression (15.00%, 35.00%, and 49.44%, respectively). Furthermore, we investigated risk factors predicting OS and cumulative recurrence of patients after hepatic resection. The univariate analysis showed that AJCC stage (*P* = 0.008, *P* = 0.005), lymphatic metastasis (*P* = 0.002, *P* = 0.002), number of tumors (*P* < 0.001, *P* < 0.001), and COTE1 overexpression (*P* < 0.001, *P* < 0.001) were risk factors for both OS and cumulative recurrences (Table 2). However, only the number of tumors (*P* < 0.001, *P* < 0.001) and COTE1 overexpression (*P* = 0.037, *P* = 0.001) was independent risk factors for both OS and cumulative recurrences according to the results of multivariate analysis (Table 2). Patients with high COTE1 expression were more likely to suffer from tumor recurrence (HR = 4.636, 95% CI = 1.943-11.062; Table 2).

### 3.3. COTE1 Mediates ICC Cell Proliferation and Clonogenicity

**Figure 2:**
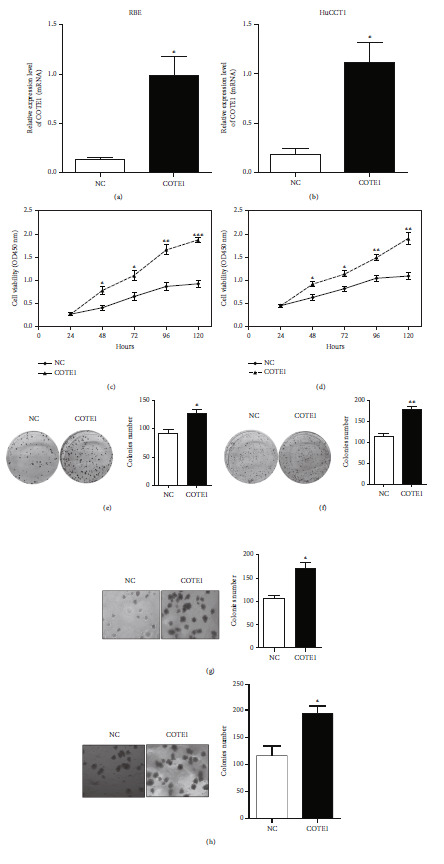
Overexpression of COTE1 promotes proliferation of RBE and HuCCT1 cells. (a, b) Overexpression of COTE1 mRNA in RBE (a) and HuCCT1 (b) with recombinant vector transfection was confirmed by qRT-PCR. (c, d) Cell viability of RBE (c) and HuCCT1 (d) with COTE1 upregulation was measured by CCK-8 assay for 5 days after transfection. (e–h) Plate colony formation and soft agar growth assays showed colony numbers of RBE (e, g) and HuCCT1 (f, h) with COTE1 overexpression. Data are recorded as mean ± SD. ^∗^*P* < 0.05, ^∗∗^*P* < 0.01, and ^∗∗∗^*P* < 0.001.

**Figure 3:**
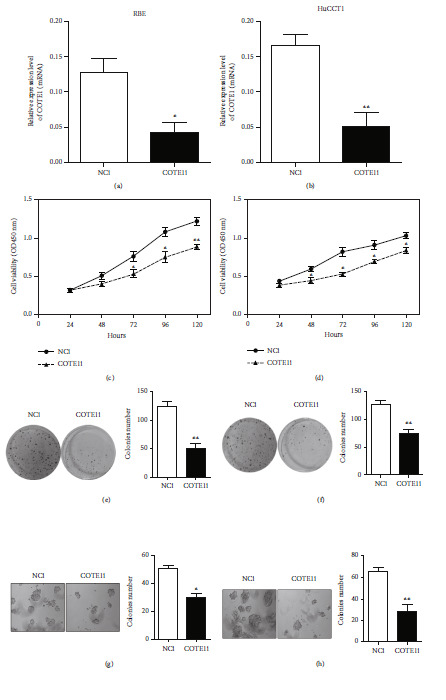
COTE1 knockdown inhibits ICC cell proliferation. (a, b) Downregulation of COTE1 mRNA in RBE (a) and HuCCT1 (b) with siRNA/shRNA transfection was confirmed by qRT-PCR. (c, d) Cell viability of RBE (c) and HuCCT1 (d) with COTE1 silencing was measured by CCK-8 assay for 5 days after transfection. (e–h) Plate colony formation and soft agar growth assays showed colony numbers of RBE (e, g) and HuCCT1 (f, h) with COTE1 knockdown. Data are presented as mean ± SD. ^∗^*P* < 0.05 and ^∗∗^*P* < 0.01.

To probe the effect of COTE1 overexpression on proliferation of ICC cells, we transfected the recombinant vector containing COTE1 into RBE and HuCCT1 cells and confirmed the overexpression of COTE1 by qRT-PCR (*P* < 0.05; Figures 2(a) and 2(b)). CCK-8 assays showed that ectopic COTE1 expression significantly facilitated the growth of these cells (Figures 2(c) and 2(d)). To further explore the long-term effect of COTE1 on cellular viability, stable cells with COTE1 overexpression were used in the plate colony formation and soft agar growth assays. As shown, cells overexpressing COTE1 generated dramatically more colonies than cells expressing only vector (Figures 2(e)–2(h)). Conversely, we investigated the potential role of COTE1 knockdown on ICC cell proliferation. The chemically synthesized small interfering RNAs (siRNAs) against COTE1 were transiently transfected into ICC cell lines, and the efficiency of RNAi was validated by qRT-PCR (Figures 3(a) and 3(b)). Expectedly, the siRNA-mediated COTE1 silencing significantly suppressed the growth of ICC cells, as shown in Figures 3(c) and 3(d). Subsequently, the constructed recombinant plasmid encoding a short hairpin RNA (shRNA) against COTE1 was used to knock down endogenous COTE1, and the influence of silenced COTE1 on colony formation was evaluated by colony-forming assays. As presented, the number of colonies in COTE1 knockdown cells was much less than that in controls (Figures 3(e)–3(h)). These data demonstrated that regulation of COTE1 expression could clearly mediate proliferation and clonogenicity of RBE and HuCCT1 cells.

### 3.4. COTE1 Influences the Invasion and Migration of ICC Cells

**Figure 4:**
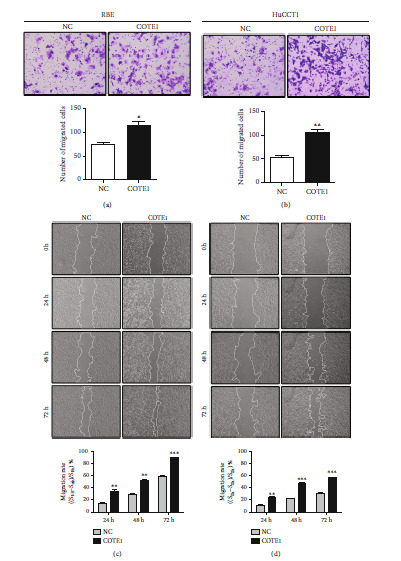
COTE1 overexpression facilitates invasion and migration of RBE and HuCCT1 cells. RBE and HuCCT1 cells were transfected with the recombinant vector containing COTE1 for 72 h. (a, b) Comparison of the invasive cells of RBE (a) and HuCCT1 (b). (c, d) Wound-healing assay comparing the motility of RBE (c) and HuCCT1 (d) cells. The wound-healing area was analyzed using the ImageJ software; the migration rate was measured using the following formula: [(wounded area 0 h − wounded area n h)/wounded area 0 h] × 100% (*n* = 24, 48, and 72). Data are recorded as mean ± SD. ^∗^*P* < 0.05, ^∗∗^*P* < 0.01, and ^∗∗∗^*P* < 0.001.

**Figure 5:**
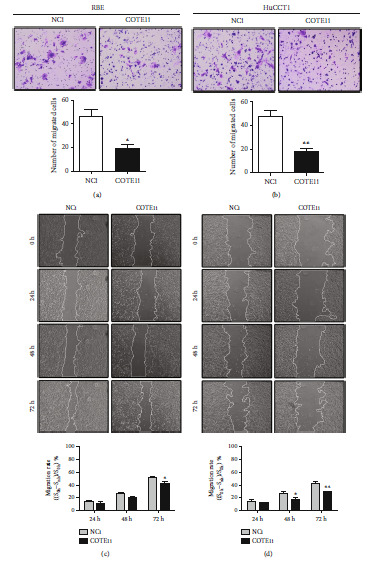
COTE1 silencing suppresses ICC cell invasion and migration. After siRNA transfection for 72 h, invasion and migration of RBE and HuCCT1 were examined by transwell and wound-healing assays. (a, b) Comparison of the invasion of RBE (a) and HuCCT1 (b) cells. (c, d) Wound-healing assay comparing the motility of RBE (c) and HuCCT1 (d) cells. The wound-healing area was analyzed using ImageJ, and the migration rate was measured using the following formula: [(wounded area 0 h − wounded area n h)/wounded area 0 h] × 100% (*n* = 24, 48, and 72). Data are recorded as mean ± SD. ^∗^*P* < 0.05, and ^∗∗^*P* < 0.01.

To determine whether COTE1 influences the invasion and migration of ICC cells, Matrigel and wound-healing assays were performed. In COTE1 overexpression cells, strong ability for invasion (Figures 4(a) and 4(b)) and higher migration rate (Figures 4(c) and 4(d)) were observed, while COTE1 knockdown reduced invasiveness (Figures 5(a) and 5(b)) and attenuated the ability of mobility (Figures 5(c) and 5(d)) in RBE and HuCCT1 cells. These collective results implied that COTE1 likely contributes to invasion and migration of ICC cells.

### 3.5. COTE1 Regulates Autophagy in ICC Cells

**Figure 6:**
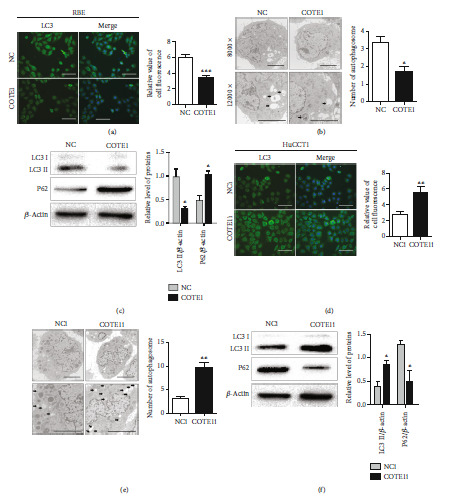
COTE1 regulates autophagy in ICC cells. RBE and HuCCT1 cells were transfected with pcDNA3.1B-COTE1 and siRNA-COTE1 for COTE1 upregulation and silencing, respectively. (a, d) Immunofluorescence (magnification, ×100; bar = 10 *μ*m) of LC3 puncta (green) in RBE (a) and HuCCT1 (d) cells. The relative value of cell fluorescence was analyzed by the ImageJ software. (b, e) TME (magnification, up image ×12000, low image, ×20000; bar = 500 nm) showed autophagosome formation (black arrows) in RBE (b) and HuCCT1 (e) cells. (c, f) Autophagy-related proteins (LC3II, P62) were detected by WB in RBE (c) and HuCCT1 (f) cells. The intensity of protein bands was analyzed by the ImageJ software, and the relative COTE1 levels were normalized to *β*-actin. Data are represented as mean ± SD. ^∗^*P* < 0.05, ^∗∗^*P* < 0.01, and ^∗∗∗^*P* < 0.001.

Since the similar contribution to carcinogenesis of COTE1 was found in both RBE and HuCCT1 cells, we used RBE cells for COTE1 overexpression and HuCCT1 cells for COTE1 knockdown, respectively, in subsequent experiments. Because of close correlation between autophagy and oncogenesis, we examined that the autophagy in ICC cells to define the potential molecular mechanisms of COTE1 contributes to ICC progress. Autophagy was detected by immunofluorescence (IF), transmission electron microscopy (TME), and western blotting (WB). As shown, COTE1 overexpression suppressed autophagy of RBE cells, which was proved by reduced intensity of LC3 fluorescence (Figure 6(a)), decreased number of autophagic vesicles (Figure 6(b)), and downregulated expression of LC3 II and P62 proteins (two main indicators of autophagic process, Figure 6(c)). On the contrary, COTE1 silencing triggered autophagy of HuCCT1 cells: more LC3 puncta accumulation (Figure 6(d)), increased autophagosome formation (Figure 6(e)), and raised LC3 II and P62 expression level (Figure 6(f)). Taken together, these data suggested that COTE1 may modulate autophagy in ICC cells, potentially providing insight into the biological mechanisms responsible for the occurrence and development of ICC.

### 3.6. COTE1 Affects Autophagy-Related Pathways via ATG6 (Beclin1) Modulation In Vitro and In Vivo

**Figure 7:**
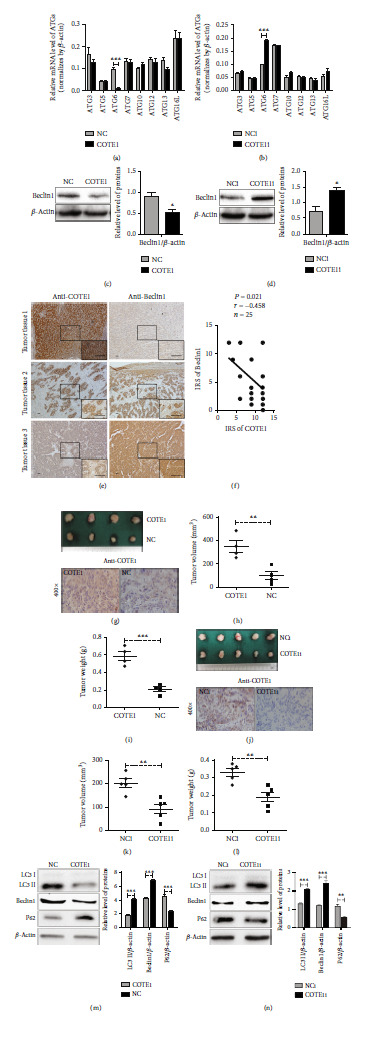
COTE1 affects autophagy-related pathways via ATG6 (Beclin1) modulation in vitro and in vivo. To upregulate/knock down COTE1 expression, RBE and HuCCT1 were transfected with pcDNA3.1B-COTE1/siRNA-COTE1, respectively. (a, b) The mRNA expression of autophagy-related genes involved in autophagy was determined by qRT-PCR in RBE (a) and HuCCT1 (b) cells. (c, d) The protein level of Beclin1 in RBE (c) and HuCCT1 (d) was detected by WB, and the intensity of bands was analyzed using ImageJ software. (e) Representative results showed COTE1 and Beclin1 protein expressed in 25 matched ICC tumor tissues (original magnification, ×100, low right image, ×400; bar = 10 *μ*m). (f) The correlation between COTE1 and Beclin1 in ICC specimens was investigated by Pearson's correlation analysis (*n* = 25, *r* = −0.458, *P* = 0.021). (g–i, m) Xenograft models of RBE cells with stable COTE1 overexpression showed tumor volume (g, h), tumor weight (i), and protein level of LC3 II, Beclin1, and P62 (m). (j–l, n) The tumor volume (j, k), tumor weight (l), and autophagy-related proteins' (LC3 II, Beclin1, and P62, n) expression in HuCCT1 cells with COTE1 stable knockdown xenograft tumors. The protein bands were quantized by ImageJ. Data are recorded as mean ± SD. ^∗^*P* < 0.05, ^∗∗^*P* < 0.01, and ^∗∗∗^*P* < 0.001.

Autophagy is a highly dynamic metabolic process, and the accumulation of autophagosomes is tightly regulated by a limited number of autophagy-related genes (ATGs) [25]. In order to identify which ATGs are involved in modulation of autophagy by COTE1, we performed qRT-PCR to investigate the expression of ATG in ICC cells following overexpression/inhibition of COTE1 [26]. Fortunately, the mRNA expression of Beclin1 (the mammalian ortholog of yeast ATG6) was clearly downregulated by COTE1 overexpression in RBE cells (Figure 7(a)). In contrast, the upregulated mRNA expression of Beclin1 was found in COTE1 silencing HuCCT1 cells (Figure 7(b)). Besides, the similar results were obtained in the analyses of Beclin1 protein expression by performing WB assay in correspondingly treated ICC cells (Figures 7(c) and 7(d)). To consolidate the notion that COTE1 affects autophagy-related pathways via Beclin1 modulation, we further analyzed the correlation between COTE1 and Beclin1 in clinical tumor specimens. As expected, COTE1 protein expression (19/25) was negatively correlated with Beclin1 expression (*n* = 25, *r* = −0.458, *P* = 0.021; Figures 7(e) and 7(f)).

To extend these results to an in vivo context, we established offspring subclones with stable COTE1 overexpression/knockdown in RBE and HuCCT1 cells, respectively, and injected these stable subclones subcutaneously into athymic mice for xenograft model construction. After monitoring for 28 days, the volume and weight of tumors formed in RBE cells overexpressing COTE1 were higher than those of tumors formed from the controls (Figures 7(g)–7(i)); inversely, COTE1 knockdown inhibited the in vivo tumorigenicity of HuCCT1 cells, as shown by the reduced size and weight of xenograft tumors (Figures 7(j)–7(l)). The presence of COTE1 in these tumors was confirmed by histological analysis. Subsequently, the autophagy-related proteins including LC3 II, Beclin1, and P62 were measured in these tumors. We observed that ectopic COTE1 overexpression caused depletion of LC3 II and Beclin1 and increasing of P62 (Figure 7(m)), while COTE1 silencing led to LC3 II, Beclin1 elevation, and P62 degradation (Figure 7(n)). The above data suggested that COTE1 could affect autophagy of ICC cells through regulating Beclin1 expression in vitro and in vivo.

### 3.7. COTE1 Knockdown Inhibits ICC Cell Progression via Beclin1-Dependent Autophagy Regulation

**Figure 8:**
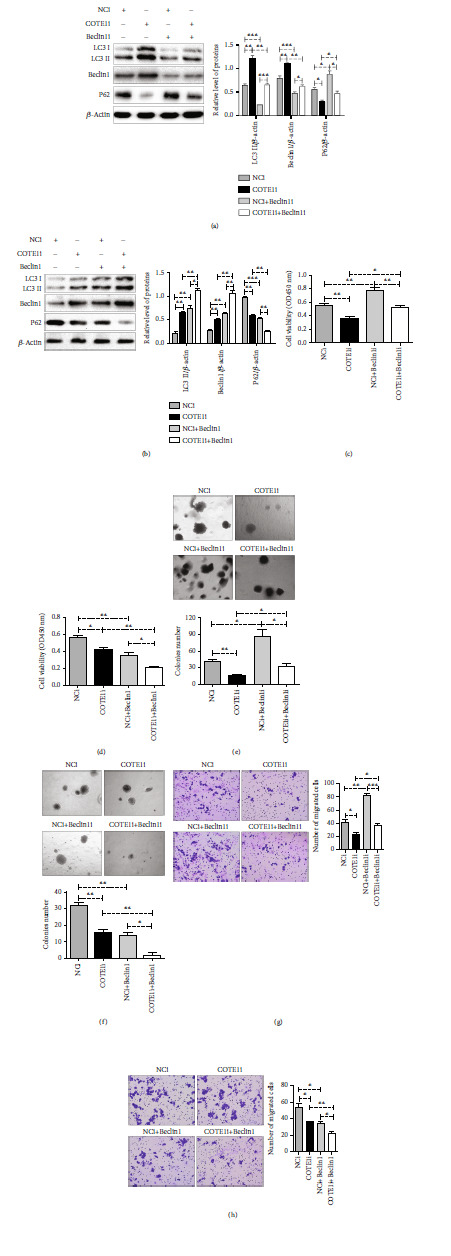
COTE1 knockdown inhibits ICC cell progression via Beclin1-dependent autophagy regulation. The offspring subclones with stable COTE1 knockdown in HuCCT1 cells were transfected with pcDNA-Beclin1 or siRNA-Beclin1. (a, b) Western blotting analysis of LC3 II, Beclin1, and P62 protein expression. The intensity of protein bands was analyzed by ImageJ, and the level of proteins was normalized to actin. (c, d) The cell viability of HuCCT1 was measured by CCK-8 assay. (e, f) The soft agar assay of colony formation. (g, h) Comparison of invasion of HuCCT1 cells using transwell compartments. Data are recorded as mean ± SD. ^∗^*P* < 0.05, ^∗∗^*P* < 0.01, and ^∗∗∗^*P* < 0.001.

Since the above data indicate that COTE1 facilitates cellular proliferation and invasion and affects autophagy through Beclin1 modulation, a hypothesis was proposed that the oncogenic role of COTE1 affecting ICC progression is regulated by Beclin1-dependent autophagy. To test this hypothesis, we modulated the expression level of Beclin1 in stable ICC cells and observed the corresponding influences on expression of autophagy-related proteins and malignant biological properties of those cells. Because of LC3 II and Beclin1 proteins were hardly detected in COTE1 upregulation RBE stable cells after Beclin1 silenced by siRNA (data not shown), we performed the following experiments in stable HuCCT1 cells with COTE1 knockdown. As expected, Beclin1 inhibition restrained autophagy induced by COTE1 silencing (LC3 II and Beclin1 reduction, P62 elevation; Figure 8(a)), whereas autophagy was enhanced after Beclin1 was restored in HuCCT1 cells (increased LC3II and Beclin1, reduced P62; Figure 8(b)). Moreover, the suppression of COTE1 knockdown on cellular growth and invasion was rescued by Beclin1 inhibition (Figures 8(c), 8(e), and 8(g)); conversely, this suppressive effect was aggravated by Beclin1 accumulation (Figures 8(d), 8(f), and 8(h)), which was validated by CCK-8, soft-agar colony formation, and cellular invasion assays, respectively. In conclusion, these findings supported the notion that COTE1 knockdown may inhibit ICC cell progression through Beclin1-dependent autophagy regulation.

## 4. Discussion

**Figure 9:**
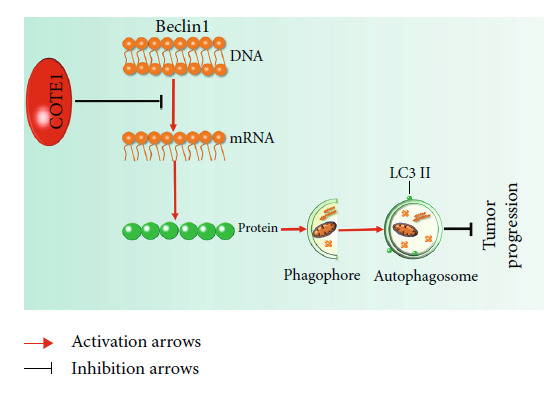
Schematic diagram for the mechanism of COTE1 contributes to ICC progression via Beclin1-dependent autophagy modulation. In ICC cells, ectopic COTE1 overexpression inhibits autophagy-related gene Beclin1 expression, which in turn induce autophagy suppression, subsequently resulting in tumor progression inhibition.

The amplification of 1q21 target genes commonly causes oncogenic phenotypes in multiple malignant tumors [18, 27, 28]. In the present study, the 1q21 mapped COTE1 gene was reconfirmed to be an oncogene in ICC, which was consistent with the results in our earlier research in HCC [22]. We found that COTE1 expression was clearly increased in ICC tissues compared with paracarcinoma tissues. Patients with high expression of COTE1 seem to have aggressive tumor features, such as increased CEA level, poorly histologic differentiation, lymphatic metastasis, vascular invasion, and multiple tumors. Furthermore, COTE1 expression in ICC was an independent predictor of the OS and recurrence of ICC patients; patients with overexpressed COTE1 have lower OS and higher recurrence rate than those with downregulated COTE1 expression. Based on the experiments on COTE1 expression regulation in ICC cells, ectopic COTE1 overexpression has been shown to potently facilitate cellular proliferation in vitro and in vivo, promote cellular invasion and migration in vitro, and inhibit autophagy. By contrast, COTE1 knockdown suppressed cell viability in vitro and in vivo, attenuate the ability of mobility in vitro, and enhance autophagy. Besides, we uncovered a novel mechanism of COTE1 in regulating autophagy via the expression of Beclin1 modulation in vitro and in vivo. Beclin1 upregulation/silencing in COTE1 knockdown stable cells augmented/attenuated LC3 II processing and P62 degradation, which in turn induced corresponding influences on ICC cell proliferation and invasion. These data suggest that COTE1 promotes ICC progression through Beclin1-dependent autophagy regulation, as illustrated in schematic diagram in Figure 9.

Evidences suggested that autophagy appears to have a dual role in cancers, including ICC [29, 30]. As O'Dell et al. [31] and Huang and Hezel [32] reported, autophagy was evaluated in ICC cells and murine model, and chloroquine-induced autophagy inhibition suppresses the accumulation of LC3 II and growth of these cells. These data indicate that autophagy plays an active role in tumor progression. Conversely, autophagy is likely to be a tumor suppressor in ICC cells. Lendvai et al. [33] discovered that autophagy activity in ICC tumor tissues was inhibited. Wang et al. [34] confirmed that the inhibition of pterostilbene on viability, migration, and proliferation of ICC cells was dependent on autophagy induction. Interestingly, in our study, the suppressive role of autophagy on proliferation and invasion was potently verified by regulating COTE1 expression in ICC cells.

The pathway of autophagy is tightly mediated by a series of autophagy-related genes (ATGs), which were confirmed to participate in tumor development of various cancers [25, 35]. In our current study, Beclin1, the mammalian homolog of yeast ATG6, was found lowly expressed in clinical tumor tissues (19/25) and negatively associated with COTE1, implying its suppressive role and potential correlation with oncogenes in ICC. Our results of Beclin1 deficiency in ICC specimens were similar to previous reports [36, 37] but were opposite to that of Bi et al. reported [38]. Additionally, in our research, Beclin1 could be regulated by COTE1 modulation in ICC cells, resulting in autophagy alteration, which in turn contribute to cellular growth and invasion of ICC. The result of negative regulation of oncogene COTE1 on Beclin1 in our experiment was consistent with the findings of Han et al. [12]; the oncogene TRIM59 could negatively regulate autophagy via modulating both the transcription and the ubiquitination of Beclin1 in non-small-cell lung cancer (NSCLC). However, Zhang et al. [13] observed that oncogenic protein COPS3 can interact with Beclin1, proved by coimmunoprecipitation, and positively regulate Beclin1 level, which subsequently induce metastasis inhibition of osteosarcoma. These above contradictory results of autophagy and Beclin1 in ICC indicated that, even in the same tumor, due to the heterogeneity and molecular aberrations distinct [39], the characteristics of autophagy might be distinguished among the various cell strains, different developmental stages, and diverse contexts [40], suggesting more molecular biomarker identification may be benefit for better understanding the intrinsic mechanism of autophagy in ICC.

In our previous studies [21, 22], we discovered COTE1 facilitated progression of hepatocellular carcinoma via WW domain containing oxidoreductase- (WWOX-) mediated cell cycle, apoptosis, and cellular invasion modulation. Currently, the oncogene COTE1 was found to promote cell proliferation and invasion of ICC by regulating Beclin1-dependent autophagy. However, the internal mechanism remains unclear. Firstly, the autophagy-dependent cell cycle and death were not investigated in this study. Recently, autophagy was reported to play an important regulatory role in cell cycle and death in cancer cells [41, 42]. Wu et al. [43] found an inverse correlation between autophagy and cyclin D1, one checkpoint of cell cycle, in hepatocellular carcinoma, and activated autophagy could selectively degrade cyclin D1, which in turn suppressing cell proliferation via the cell cycle arrest at the G1 phase. Besides, in pancreatic cancer, Ye et al. [44] provided evidence of ferroptosis, a type of autophagy-dependent cell death, which could potentiate cytotoxic effect of gemcitabine. Unfortunately, the cell cycle and death of ICC in our recent experiments were not detected, which may need further investigation. Secondly, autophagy was considered as a regulator of TGF-*β*-induced epithelial-mesenchymal transition (EMT) and invasion [45]. Although we confirmed the effect of autophagy on cellular migration and invasion in ICC in this study, the molecular mechanism is indistinct. Thirdly, the regulation of COTE1 on Beclin1 is superficial, lacking evidences of their direct or indirect interaction. Lastly, in HCC, we found coprecipitation of protein COTE1 and WWOX [22], a classical tumor suppressor which was also lost/reduced expression in ICC [46, 47]; whether the similar phenomenon exists in ICC cells is still uncertain. To sum up, these potential clues imply us for further investigation of COTE1 in ICC.

In conclusion, we have, for the first time, demonstrated that COTE1 is upregulated in ICC patients, and overexpression of COTE1 is associated with aggressive clinical features and predicts poor prognosis of ICC patients. Functionally, we revealed that ectopic COTE1 expression could facilitate cell proliferation, tumorigenesis, and invasion through Beclin1-dependent autophagy regulation. To our knowledge, these findings provide attractive new option for understanding underlying mechanism of ICC progression and generate novel opportunities for its future treatment.

## 5. Conclusions

In this study, our results indicated that COTE1 is an oncogene in ICC pathogenesis, and the ectopic COTE1 expression promotes ICC proliferation and invasion via Beclin1-dependent autophagy inhibition.

## Figures and Tables

**Table 1:** Correlation of COTE1 and clinical characteristics of ICC.

Clinical characteristics	COTE1 expression		*P*
	High	Low	
Gender	25	8	0.059
	13	12	
Age	32	15	0.395
	6	5	
HBsAg	8	2	0.290
	30	18	
HCV^&^	4	1	0.650
	34	19	
CA199	5	5	0.256
	33	15	
CEA^&^	0	3	*0.037*
	38	17	
AFP	5	3	0.847
	33	17	
AJCC tumor stage	12	2	0.068
	26	18	
Histologic differentiation	23	4	*0.003*
	15	16	
Tumor size	22	7	0.097
	16	13	
Lymphatic metastasis	18	0	<*0.001*
	20	20	
Vascular invasion	28	8	*0.012*
	10	12	
Number of tumors	8	0	*0.027*
	30	20	

^&^Fisher's exact test. Values in italics indicate statistical significance.

**Table 2:** Univariate and multivariate analyses of factors associated with survival and recurrence.

Variables	OS			
	Univariate *P*	Analysis HR (95% CI)	Multivariate *P*	Analysis HR (95% CI)
Gender (male vs. female)	0.135	1.569 (0.869-2.832)		
Age (>60 vs.≦60 year)	0.136	1.873 (0.821-4.271)		
HBsAg (positive vs. negative)	0.454	1.322 (0.638-2.748)		
HCV (positive vs. negative)	0.255	1.742 (0.670-4.532)		
CA19-9 (>37 vs. ≦37 U/mL)	0.433	1.454 (0.571-3.704)		
CEA (>5.29 vs. ≦5.29 *μ*g/L)	0.636	0.752 (0.231-2.449)		
AFP (>7.29 vs. ≦7.29 *μ*g/L)	0.761	1.133 (0.506-2.537)		
AJCC tumor stage (III-IV vs. I-II)	*0.008*	2.410 (1.263-4.601)		
Histologic differentiation (III-IV vs. I-II)	0.181	1.481 (0.833-2.630)		
Tumor size (>5 vs. ≦5 cm)	0.203	1.456 (0.817-2.594)		
Lymphatic metastasis (yes vs. no)	*0.002*	2.739 (1.453-5.165)	0.073	1.910 (0.941-3.874)
Vascular invasion (yes vs. no)	0.276	1.411 (0.760-2.620)		
Number of tumors (multiple vs. single)	<*0.001*	8.559 (3.434-21.333)	<*0.001*	7.241 (2.750-19.067)
COTE1 overexpression (yes vs. no)	<*0.001*	3.987 (1.871-8.494)	*0.037*	2.528 (1.057-6.051)

Variables	Cumulative recurrence			
	Univariate *P*	Analysis HR (95% CI)	Multivariate *P*	Analysis HR (95% CI)
Gender (male vs. female)	0.069	1.752 (0.957-3.207)		
Age (>60 vs.≦60 year)	0.376	1.425 (0.650-3.124)		
HBsAg (positive vs. negative)	0.396	1.373 (0.660-2.858)		
HCV (positive vs. negative)	0.729	1.202 (0.424-3.407)		
CA19-9 (>37 vs. ≦37 U/mL)	0.841	1.112 (0.396-3.122)		
CEA (>5.29 vs. ≦5.29 *μ*g/L)	0.785	0.848 (0.260-2.768)		
AFP (>7.29 vs. ≦7.29 *μ*g/L)	0.870	0.930 (0.393-2.200)		
AJCC tumor stage (III-IV vs. I-II)	*0.005*	2.527 (1.318-4.846)		
Histologic differentiation (III-IV vs. I-II)	0.076	1.697 (0.946-3.044)		
Tumor size (>5 vs. ≦5 cm)	0.333	1.332 (0.745-2.381)		
Lymphatic metastasis (yes vs. no)	*0.002*	2.796 (1.476-5.297)		
Vascular invasion (yes vs. no)	0.387	1.316 (0.706-2.454)		
Number of tumors (multiple vs. single)	<*0.001*	9.427 (3.646-24.372)	<*0.001*	6.399 (2.458-16.661)
COTE1 overexpression (yes vs. no)	<*0.001*	5.321 (2.276-12.439)	*0.001*	4.636 (1.943-11.062)

Values in italics indicate statistical significance.

## Data Availability

The datasets generated during and/or analyzed during the current study are available from the corresponding author on reasonable request.

## Supplementary Materials

Supplementary Materials Supplementary Table S1: the sequences of primers and siRNAs/shRNAs used in this study.
