# Supplementary material for: COTE1 Facilitates Intrahepatic Cholangiocarcinoma Progression via Beclin1-Dependent Autophagy Inhibition
Source: Biomed Res Int. 2023 Sep 22;2023:5491682. doi: 10.1155/2023/5491682 (PMC10541304; doi:10.1155/2023/5491682)
Supplement: Supplementary Materials — Supplementary Table S1: the sequences of primers and siRNAs/shRNAs used in this study. [file 5491682.f1.docx]

| **Table S1. The sequences of primers and siRNAs/shRNAs used in this study** | | |
| --- | --- | --- |
| Genes/RNAs |  | Sequences |
| COTE1 | sence | 5'-GGGCTCTGACCTAGGCTTCT-3' |
|  | Antisence | 5'-ACAGAAGCTCTCCCAGTCCA-3' |
| ATG3 | sence | 5'-GACCCCGGTCCTCAAGGAA-3' |
|  | Antisence | 5'-TGTAGCCCATTGCCATGTTGG-3' |
| ATG5 | sence | 5'-AAAGATGTGCTTCGAGATGTGT-3' |
|  | Antisence | 5'-CACTTTGTCAGTTACCAACGTCA-3' |
| ATG6 | sence | 5'-AGGAACTCACAGCTCCATTAC-3' |
|  | Antisence | 5'-AATGGCTCCTCTCCTGAGTT-3' |
| ATG7 | sence | 5'-CAGTTTGCCCCTTTTAGTAGTGC-3' |
|  | Antisence | 5'-CCAGCCGATACTCGTTCAGC-3' |
| ATG10 | sence | 5'-AGACCATCAAAGGACTGTTCTGA-3' |
|  | Antisence | 5'-GGGTAGATGCTCCTAGATGTGAC-3' |
| ATG12 | sence | 5'-CTGCTGGCGACACCAAGAAA-3' |
|  | Antisence | 5'-CGTGTTCGCTCTACTGCCC-3' |
| ATG13 | sence | 5'-ATTTCAGAACCCCCCTCAGC-3' |
|  | Antisence | 5'-TCATGCACAGCCAGCTTCTC-3' |
| ATG16L | sence | 5'-AAGAAACGTGGGGAGTTAGC-3' |
|  | Antisence | 5'-AGAGACAGAGCGTCTCCCAA-3' |
| β-ACTIN | sence | 5'-AGAGCCTCGCCTTTGCCGATCC-3' |
|  | Antisence | 5'-CTGGGCCTCGTCGCCCACATA-3' |
| siCOTE1 | sence | 5'-GTATGTAAGCCTTCAATAA-3' |
|  | Antisence | 5'-TTATTGAAGGCTTACATAC-3' |
| siBeclin1 | sence | 5'-GATTGAAGACACAGGAGGC-3' |
|  | Antisence | 5'-GCCTCCTGTGTCTTCAATC-3' |
| shCOTE1 | sence | 5'-GATCCCCGTATGTAAGCCTTCAATAATTCAAGAGATTATTGAAGGCTTACATACTTTTTGGAAA-3' |
|  | Antisence | 5'-AGCTTTTCCAAAAAGTATGTAAGCCTTCAATAATCTCTTGAATTATTGAAGGCTTACATACGGG-3' |
| shBeclin1 | sence | 5'-GATCCCCGATTGAAGACACAGGAGGCTTCAAGAGAGCCTCCTGTGTCTTCAATCT TTTTGGAAA-3' |
|  | Antisence | 5'-AGCTTTTCCAAAAAGATTGAAGACACAGGAGGCTCTCTTGAAGCCTCCTGTGTC TTCAATCGGG-3' |
